# Epigenetic Dysregulations in Arsenic-Induced Carcinogenesis

**DOI:** 10.3390/cancers14184502

**Published:** 2022-09-16

**Authors:** Ranakul Islam, Lei Zhao, Yifang Wang, Grace Lu-Yao, Ling-Zhi Liu

**Affiliations:** Department of Medical Oncology, Sidney Kimmel Cancer Center, Thomas Jefferson University, Philadelphia, PA 19107, USA

**Keywords:** epigenetic regulation, arsenic, carcinogenesis, angiogenesis

## Abstract

**Simple Summary:**

Arsenic is a chemical element that is toxic, and long-term exposure to it causes cancers such as lung, skin, liver, and bladder cancers. Over 150 million people around the world are affected by arsenic exposure. However, the molecular mechanism of how arsenic induces carcinogenesis is still not clear. As a carcinogen, arsenic has been demonstrated not to cause point mutations. Hence, the understanding of the dysregulation of epigenetic mechanisms caused by arsenic may help to unravel the mechanisms by which arsenic induces cancers.

**Abstract:**

Arsenic is a crucial environmental metalloid whose high toxicity levels negatively impact human health. It poses significant health concerns to millions of people in developed and developing countries such as the USA, Canada, Bangladesh, India, China, and Mexico by enhancing sensitivity to various types of diseases, including cancers. However, how arsenic causes changes in gene expression that results in heinous conditions remains elusive. One of the proposed essential mechanisms that still has seen limited research with regard to causing disease upon arsenic exposure is the dysregulation of epigenetic components. In this review, we have extensively summarized current discoveries in arsenic-induced epigenetic modifications in carcinogenesis and angiogenesis. Importantly, we highlight the possible mechanisms underlying epigenetic reprogramming through arsenic exposure that cause changes in cell signaling and dysfunctions of different epigenetic elements.

## 1. Arsenic and Mechanisms of Arsenic-Induced Carcinogenesis

Arsenic (As), a chemical element, is classified as a toxic metalloid and is associated with various human cancers [1], and its toxicity depends on the molecular form and oxidation state. International Agency for Research on Cancer (IARC) and US Environmental Protection Agency (USEPA) designated arsenic as Group 1 and Group A human carcinogens, respectively [2,3]. Furthermore, it is graded as first on the substance priority list in the Agency for Toxic Substances and Disease Registry (ASTDR), USA (https://www.atsdr.cdc.gov/spl/index.html, accessed on 9 February 2022) [4]. Chronic exposure to dietary arsenic is linked to skin, bladder, liver, and lung cancer [4,5,6,7]. Drinking water contaminated with arsenic has been linked with increased mortality of both noncancerous diseases and cancers in Bangladesh [8]. Both chronic and acute exposure to arsenic is harmful to different tissues and organs in the body, such as alteration in skin pigmentation and hyperkeratosis, peripheral neuropathy, development, cognitive impairments, and cardiovascular diseases. 

The contamination of arsenic increases with the finding of newer places [9]. The familiar sources of arsenic exposure include drinking water, food, and inhalation in an industrial work setting. Over 150 million people on the earth are exposed to carcinogenic (10 μg/L) levels of arsenic [9,10], and the majority of these people are affected by drinking water from aquifers contaminated with arsenic. Countries with arsenic concentrations exceeding this carcinogenic level (10 μg/L) in the drinking water include Bangladesh, India, China, Argentina, Mexico, Canada, the USA, and Chile [11]. Arsenic exposure to foods usually occurs by growing crops in the soil contaminated with arsenic and/or irrigating water contaminated with arsenic [12]. Furthermore, NIOSH estimates that approximately 1.5 million workers have been affected by arsenic or arsenic compounds [13].

Arsenic has several states; the most common valence states of arsenic are inorganic As^III^ (arsenite) and As^V^ (arsenate). Inorganic arsenic is very toxic to humans, whereas organic arsenic has low toxicity. As^III^ is the highest toxic form because it is more soluble in water than arsenic compounds. It contains a lone electron pair that can engage in chemical bonds [14,15]. Depending on the types of food, arsenic can be found in both inorganic (when combined with oxygen, chlorine, and sulfur, among other elements) and organic forms (when linked with carbon and hydrogen). Inorganic arsenic is typically found in the inorganic form in drinking water, soil, and some terrestrial foods such as rice, as either As^III^ or As^V^. Inorganic pentavalent arsenic As^V^ is absorbed by the body through drinking water and uses membrane transporters such as aquaporin and inorganic phosphate transporters (PiT) to enter the cells [16,17]. In the cell, arsenic As^V^ is converted to the more toxic form arsenite in a glutathione-dependent reaction (GSH), with subsequent methylation to mono-methylated (MMA) and di-methylated arsenicals (DMA), respectively [18,19]. Methylated arsenicals, especially MMA^III^, are considered more toxic than inorganic As^III^ both in vivo (in hamsters) [20] and in vitro (human cell lines) [21].

**Figure 1:**
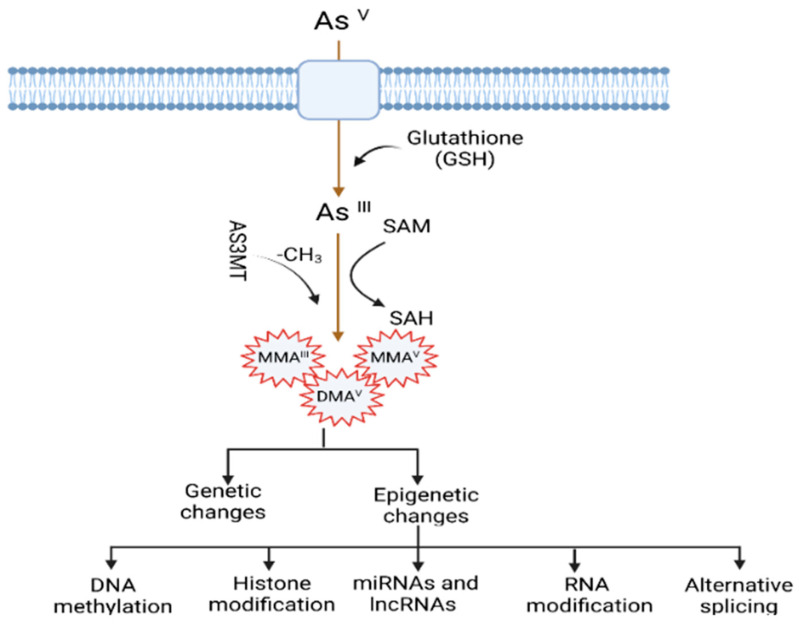
Mechanisms of arsenic-induced carcinogenesis. Arsenic exposure induces carcinogenesis via its biotransformation process, which causes effects on both genetic and epigenetic levels. The biotransformation of arsenic happens via a series of reactions such as reduction, oxidation, and methylation. Pentavalent arsenic (As^V^) is reduced to trivalent (As^III^) and then methylated into organic arsenic species with higher carcinogenic potential. Here, S-adenosylmethionine (SAM) acts as a methyl donor, and Glutathione (GSH) and other thiols serve as reducing agents. Epigenetic alterations induced by arsenic exposure include abnormal changes in DNA methylation, histone modification, miRNAs and lncRNAs expression, RNA modification, and alternative splicing.

The mechanisms by which arsenic induces carcinogenesis are still a point of debate. However, it has been known that arsenical compounds contribute to carcinogenesis by disrupting the signaling cascade, changing gene expression, elevating levels of oxidative stress and inflammation, increasing genotoxic and DNA damage, decreasing DNA repair, inducing cell cycle arrest and apoptosis [19,22,23,24], acting as co-carcinogenesis with other environmental toxicants [25], and alterations of epigenetic regulation. There is also doubt about whether arsenic is genotoxic or not because arsenic does not cause point mutations in standard mutagenicity assays; hence it is considered to be nongenotoxic [26,27]. Although arsenic is viewed as a carcinogen, its non-mutagenic characteristics violet its function in causing genetic alteration. However, a less studied mechanism, but one that is crucial for understanding arsenic-induced carcinogenesis, is the dysregulation of epigenetic modifications. Studies investigating epigenetic regulation changes upon arsenic exposure during the last decade are increasing. The researchers have attempted to explore the role of DNA methylation, histone modification, miRNAs and lncRNAs alteration, mRNA modification, and alternative splicing in arsenic toxicity and carcinogenesis. The present review will comprehensively discuss the epigenetic regulations involved in gene expression, and their dysregulation is pivotal in arsenic-induced transformation, tumor growth, and angiogenesis. The general scheme of the mechanism of arsenic-induced carcinogenesis is shown as follows (Figure 1).

## 2. Arsenic-Induced Changes in DNA Methylation

DNA methylation is the inclusion of the methyl group (-CH3) in the 5-carbon on the cytosine residues (5 mC) in CpG (Cytosine-Phosphate-Guanine) and non-CpG (CpA, CpT, and CpC) dinucleotides. The methyl group comes from a methyl donor, generally from S-adenyl methionine (SAM), and this process is mediated by DNA methyl transferases (DNMTs) [28]. CpG dinucleotides are concentrated in CpG islands (short CpG-rich DNA stretches) and regions of repetitive sequences such as centromeric repeats, retrotransposon elements, rDNA, etc. [29,30,31]. In cancers, the changes of methylation status mainly occur within CpG islands, which occupy ~70% of all mammalian promotors. In addition, these islands play an important role in the regulation of transcription, and their general changes have been found during malignant transformation [32,33]. The functional effect of the dysregulation of DNA methylation is context- and spatial-dependent, dynamic, tissue-specific, and trans-generationally heritable [34,35,36]. Generally, gene silencing involves promotor methylation, and constitutive gene expression is associated with gene body methylation [32]. However, the methylation of the gene body may also be found to inactivate repetitive DNA elements within the gene body [35,37] and show dramatic alteration intron-exon boundaries [38]. These complex methylation patterns underline the necessity of DNA methylation profiling to answer biological questions.

Although it is evident that the dysregulation of DNA methylation has been demonstrated in different cancers, our knowledge of the impact of inorganic arsenic (iAs) on DNA methylation is still growing. Methyl transferase (MTs) catalyze the methyl group transfer in the 5-carbon on the cytosine residues (5 mC) in CpG dinucleotides and use SAM, a coenzyme, as a methyl group donor. Long-term exposure to arsenic causes depletion of the SAM by MTs such as AS3MT [arsenic (III) methyl transferase]. Furthermore, arsenic can also control DNMTs and decrease their activities. For example, studies have found that arsenic exposure causes a reduction of mRNA levels and activity of DNMTs [39,40,41].

iAs exposure has been shown to change global DNA methylation in vitro, in animal studies as well as in population studies (Table 1). For instance, a chronic low-dose of iAs exposure induces DNA hypo-methylation in cells [42]. In addition, fish, mice, and rats exposed to iAs exhibit hepatic global DNA hypomethylation [42,43,44]. However, limited studies are available for the human population compared to in vitro and animal studies. A recent study assessed the association between arsenic exposure and global DNA methylation (∼850,000 CpGs) through drinking water among 396 Bangladeshi people who joined the Health Effects of Arsenic Longitudinal Study (HEALS). The study identified 34 CpGs associated with arsenic concentration in the urinary tract and found a positive relationship between higher arsenic concentration and DNA hypomethylation in those CpGs. Among the arsenic-associated CpGs, most of the genes were annotated to the reactive oxygen species (ROS) pathway, tumor necrosis factor-α (TNF-α) signaling, and inflammatory response via nuclear factor kappa B (NF-κB). These are essential hallmarks of cancer and aging [45]. The results are consistent with earlier studies indicating that epigenetic alterations potentially regulate arsenic toxicity [45]. Pilsner et al. showed that iAs exposure led to global hypomethylation of leukocytes in human skin. The authors observed that people with hypo methylation in the peripheral blood lymphocytes (PBL) DNA were prone to skin lesions two years later when they adjusted for age, urinary As, and other factors [46]. A whole-genome microarray-based study showed that the status of DNA methylation changed over time in people who were affected by arsenic-induced skin lesions compared to control in Bangladesh. The study found the top 20 differentially methylated CpG sites. Among these top CpG sites, the methylation percentages increased in 13 CpGs, and decreased in 7 CpGs between baseline and follow-up [47]. Bandyopadhyay et al. evaluated the association of cytogenetic damage by measuring lymphocyte micronucleus (MN) frequency and long interspersed nuclear element-1 (LINE-1) methylation status among children who were exposed to arsenic in the areas of West Bengal. They observed that a high reduction of LINE-1 methylation was associated with MN frequency in exposed children compared to unexposed children, suggesting that LINE-1 methylation is a potential epigenetic marker for arsenic toxicity in individuals [48]. 

Besides the changes in global DNA methylation status, iAs exposure also causes changes in DNA methylation in specific regions of targeted genes in different cancers [49]. For example, the association between arsenic exposure and hypomethylated or hypermethylated promotors of some genes was found in human skin cancer [50] and bladder cancer [51,52]. The carcinogenesis can occur due to the silence of tumor suppressor genes via hypermethylation [40]. Some studies have found that iAs exposure leads to increased methylation of the promotor for tumor suppressor genes such as *p15*, *p16*, *p53*, and *death-associated protein kinase (DAPK)* in vitro and in vivo [40,50,51], DNA repair-related genes such as *ERCC2*, *RPA1* in human hepatocytes [53], *MLH1* in whole human blood [54], and genes associated with the Wnt pathway like *MYC* and *WNT2B* [53]. However, another study involving a human population chronically exposed to arsenic demonstrated hypomethylation at the promoter of the DNA repair gene *ERCC2* [55]. Smeester et al. comprehensively studied the status of DNA methylation within CpG islands for more than 14,000 genes among arsenic-exposed individuals with skin lesions and without skin lesions [56]. They identified 183 genes with differentially methylated CpG islands, of which 182 were hyper-methylated in individuals with signs of arsenicosis. Gene enrichment analysis showed that most genes involved cancer-linked pathways via genes such as *p53*. They also identified an arsenic-methylated tumor suppressorome, a complex of 17 known or putative tumor suppressors silenced in human cancers, which includes hypermethylated genes such as chromosome 11 open reading frame 70 (*C11orf70*), centromere protein E (*CENPE*), forkhead box F1 (*FOXF1*), homeobox B5 (*HOXB5*), homeobox B9 (*HOXB9*), *hsa-mir-126*, SWI/SNF related, matrix associated, actin dependent regulator of chromatin subfamily d member 2 (*SMARCD2*), T-box brain 1 (*TBR1*), etc. Chanda et al. showed the hypermethylation of *GMDS* gene fragments in the peripheral blood leukocyte DNA of individuals exposed to arsenic and with skin cancer. They indicated it as a biomarker for arsenic-induced cancer [57]. The *AS3MT* gene plays an essential role in the metabolism of arsenic and its toxicological mechanism. Gribble et al. found decreased methylation in the promotor region of *AS3MT* in an arsenic-exposed area in Arizona [58]. However, no further studies have been performed to investigate the association between skin lesion status and *AS3MT* promoter methylation to date. On the other hand, carcinogenesis can also occur due to the activation of oncogene genes via hypomethylation. For instance, mice treated with iAs showed hypomethylation of the promoter region of oncogene *Hras1* and increased mRNA levels of *Hras1* [59], which was consistent with another study showing hypomethylation and increased mRNA levels of *Hras1* and *c-myc* in vitro [60,61]. Arsenic exposure also led to *Esr1* gene overexpression via hypomethylation of its promoter region, which is closely related to arsenic-induced hepatocarcinogenesis [44]. However, a recent study by Janasik et al. found hypermethylation of genes promoter of Nuclear factor-erythroid factor 2-related factor 2 (*NRF2*) and Kelch-like ECH-associated protein 1 (*KEAP1*) among occupationally arsenic-exposed copper mill workers from Poland [62]. 

DNA methylation inhibition occurs in a site-specific manner by proteins known as the ten-eleven translocation (TET) enzymes [63,64]. These TET enzymes oxidize 5 mc to 5 hyrdoxymethylcytosine (5 hmc). Disruption of this group of proteins has been shown in different types of cancer. Wang et al. showed that As inhibited the TET-mediated DNA demethylation and subsequently induced the hypermethylation in the promotor region to suppress the antioxidant genes 8-oxoguanine DNA glycosylase (*OGG1*) and glutathione S-transferase Pi 1 (*GSTP1*), thus increasing oxidative stress in human bronchial epithelial (HBE) cells in vitro [65]. In another recent study, Domingo-Relloso et al. conducted an epigenome-wide association study (EWAS) to compare the association of different As exposure levels and human blood 5 mc and 5 hmc markers in two diverse populations from the Aragon Workers Health Study (AWHS, Spain) and the Folic Acid and Creatinine Trial (FACT, Bangladesh) [66]. The effect of As on site-specific 5 mC and 5 hmC was measured using the Illumina methylation EPIC array on more than 850,000 CpG sites. They indicated different epigenetic effects for low As exposure in the AWHS population and high As exposure in the FACT population. The differentially methylated (DMP) and hydroxymethylated (DHP) positions were primarily found in distinct genomic sites. For example, they found three DMPs annotated to *CLEC12A*, a gene that plays a role in inflammation and immune response, which was consistent with previous studies [67]. In addition, they also found one DHP annotated to *NPLOC4*, a gene that has protein processing function in the endoplasmic reticulum (ER) in the FACT population exposed to a high dose of As. This is invariable to a study that reported a role of As in ER stress-associated protein misfolding and apoptosis [68], for which mechanisms are known to be associated with cardiometabolic diseases and cancer. 

In addition, arsenic exposure also causes transgenerational genotoxicity and the alteration of global DNA methylation patterns in the animal model. Parental chronic arsenic exposure led to genotoxic damage (F0–F3), different methylation patterns, changes in physical and reproductive parameters, abnormal morphology in the ovaries (F0 and F1) and testicles (F1–F3), and a decline in the quality of sperm (F0–F3, except F2), suggesting that an individual’s early life disruptions can negatively impact later generations’ health [36]. An association was found between low or high-dose exposure to arsenic during gestation with umbilical cord blood DNA methylation. There was increased DNA methylation in CpG sites of *LINE-1* and, to a lesser extent, within the promotor region of *p16* [69]. Studies also showed the sex-dependent association between arsenic exposure and cord blood DNA methylation status, and the impact was even more prominent in the boys than in the girls [70]. 

Arsenic doesn’t induce point mutations but causes deletion mutations and chromosomal instability [40]. One possible mechanism by which arsenical compounds contribute to carcinogenesis is the disruption of normal epigenetic marks at specific loci, which may cause changes in gene expression and carcinogenesis [71,72]. Although arsenic exposure was found to alter methylation levels in global DNA and promoters of some genes, current research is hard to understand due to the complexity and insubstantial information provided in the current studies. Further investigations are necessary to systematically explore DNA methylation on a genome-wide level in cell lines exposed to arsenic and target tissues from well-characterized arsenic-exposed populations or tumor tissues from arsenic-associated cancers. Such studies will assist in elucidating the possible biological effects of arsenic exposure on DNA methylation and carcinogenesis. Arsenic-induced alterations of DNA methylation status and carcinogenesis are summarized in Table 1.

**Table 1:** Arsenic-induced alterations of DNA methylation status and carcinogenesis.

Tissue/Cells	Source of Arsenic	DNA Methylation			References
		Global	Gene-Specific		
			Hyper	Hypo	
Prostate epithelial cell line RWPE-1	As^III^	Hypo			[73,74]
HaCaT keratinocytes	As^III^	Hypo			[39]
TRL 1215 rat liver epithelial cells	As^III^	Hypo			[42]
Goldfish	As^III^	Hypo			[75]
Fisher 344 rat	As^III^	Hypo			[43]
129/SvJ mice	As^III^	Hypo			[44]
Blood samples	Drinking water	Hypo			[45]
Blood samples from skin lesion patients and control		13 Hyper and 7 hypo methylation of CpG islands			[47]
Human		Hyper			[76]
		Hypo (in skin lesion patients)			[46]
Peripheral blood lymphocyte DNA from skin lesions and non-skin lesions	Drinking water (urine samples)		182 genes out of 183 hypermethylated; Identified a silenced tumor suppressorome consists of 17 genes		[56]
	MMA^III^		*ZHCAN12* and *C1QTNF6*		
Uroepithelial SV-HUC-1 cells	As^III^		*DAPK*		[77]
Hamster embryo cells	As^III^			*c-myc* and *Ha-ras*	[61]
TRL 1215 rat liver epithelial cells	As^III^			*c-myc*	[51]
C57BL/6J mice	As^III^			*c-Ha-ras*	[59]
A/J mice	As^V^		*p16*, *RASSF1*		[78]
C3H mice	As^III^			*ER*α	[79]
Blood samples from the people of West Bengal, India	Drinking water		*p53* and *p21* in skin cancer patients		[50]
Tissues from arsenic-induced skin lesions (cases) and with no skin lesions (controls)	Drinking water		*DAPK* and *p16*		[80]
Blood samples from copper mill workers and Non-occupationally exposed healthy controls in Poland	Copper mill (urine)		*NRF2* and *KEAP1*		[62]
Blood samples from arsenic-exposed individuals (with and without skin lesions)	Drinking water (water, urine)		*MLH1* and *MSH2*		[81]
Samples from bladder tumor	Drinking water (toenail)		*RASSF1A* and *PRSS3*		[52]
Cord blood lymphocytes	Drinking water (cord blood, nails, and hair)		*p53*		[82]
Blood samples from the West Bengal population and HEK293 cell lines	Drinking water(water, urine), sodium arsenite, As^III^			Increased *ERCC2* expression	[55]
Blood samples from arsenic-exposed individuals (with and without skin lesions)	Drinking water (water, urine)			Increased *Tfam* and *PGC1α* expression	[83]

## 3. Arsenic Alters Histone Post-Translational Modification (PTM)

Genomic DNA is vast, around 2 m in length, and fits into nuclei of approximately 6 µm diameter by packaging it into chromatin [84]. The nucleosome is the central unit of chromatin, which consists of around 146 DNA base pairs wrapped 1.65 times around an octamer of histone protein, comprising two copies of each histone: H2A, H2B, H3, and H4 [85] and linked by the fifth histone (H1), which helps stabilize the nucleosome and facilitates the folding of nucleosomes into chromatin compression [86]. Post-translational modifications (PTMs) of these histone proteins alter chromatin’s structure and function, leading to a change in gene expression. Besides chromatin structures and gene expressions, histone modifications also affect various biological processes such as replication, repair mechanisms, and the recombination of DNA [87]. Histone modification includes acetylation, methylation, phosphorylation, and others such as ubiquitylation, biotinylation, glycosylation, carbonylation, ADP-ribosylation, crotonylation, propionylation, N-formylation, sumoylation, citrullination, etc. [88]. In this review, we will discuss the most studied histone modifications due to arsenic exposure. 

### 3.1. Histone Acetylation

Histone acetylation or deacetylation is a dynamic and reversible event [89]. More than 50 years ago, Alfrey et al. demonstrated that histone acetylation and transcriptional activity were positively correlated [90]. Since then, histone acetylation has been found to be an important event for gene regulation by increasing the ability to regulate and activate transcription through chromatin modification [91]. Histone acetyltransferases (HATs) and histone deacetylases (HDACs) are the two types of antagonistic enzymes that regulate histone acetylation and deacetylation, respectively. HATs catalyzed the addition of an acetyl group to the ε-amino group of specific lysine side chains within the histone’s basic N-terminal tail region by using acetyl co-A as a donor. This event neutralizes the lysine’s positive charge and weakens the interactions between histones and DNA, resulting in a relaxation of chromatin, which favors higher transcription. The acetylation at particular lysine sites can also recruit the SWItch/Sucrose Non-Fermentable (SWI/SNF) complexes which are bromodomain-containing proteins that help change the structure of chromatin to a more open state to enable it for active transcription [92]. In contrast, HDACs remove the acetyl group from the lysine residues and favor compact chromatin [93]. 

Increasing evidence has indicated that arsenic can change the pattern of acetylation in the histone proteins at different parts of the chromatin. In the 1980s, it was reported that arsenic exposure significantly decreased histone acetylation in Drosophila [94]. More recently, alteration in both H3 and H4 histone elements has been associated with global or dose-dependent arsenic exposure [95,96,97]. For example, both As^III^ and MMA^III^ exposure has been shown to induce malignant transformation of human urothelial cells in vitro and to alter histone H3 acetylation patterns [98]. In addition, the same study found DNA hypermethylation in a number of promoters that are already hypoacetylated. This result leads us to believe that the genes may be targeted in a coordinated manner by arsenic via the alteration of various epigenetic mechanisms to promote malignant transformation [98]. In addition, H3K27 and H3K9 acetylation has been associated with occupational arsenic exposure [95,99]. During embryo development, arsenic also increases global H3K9 hypoacetylation [96]. Jo et al. showed that in human bladder epithelial cells, the H4K16 acetylation global level was decreased in a dose- and time-dependent manner after exposure to both As^III^ and MMA^III^ treatment [100]. Moreover, silencing the gene MYST1, which is needed for H4K16 acetylation, caused higher cytotoxicity from arsenical exposure, suggesting that H4K16 acetylation may be crucial for resistance to arsenic-induced toxicity. Several other researchers have investigated the influence of arsenic on specific histone acetylation and noticed dissimilarities from lysine residue. Arsenic did not change H4K5ac [99,101] or H4K8ac [101]. As^III^ did not change H3K14ac in the APL cell lines [102]. However, an epidemiological study observed a positive association between urinary arsenic and H3K14ac in lymphocytes [103]. Interestingly, As^III^ exposure was found to upregulate the genes required in apoptosis or for the response to cell stress by inducing histone acetylation via HDACs [102,104] and by hindering *HDAC* genes that associate with higher global histone acetylation [105]. It has been reported that the HDAC inhibitor can restore arsenic-induced endothelial dysfunction and dementia, and inhibit malignant transformation induced by iAs [101,106].

### 3.2. Histone Methylation 

Histone methylation takes place mainly on the lysine and arginine side chain and does not change the charge of the histone protein, as opposed to histone acetylation and phosphorylation. Furthermore, this modification adds another level of complexity. Lysine can be mono-, di-, or tri-methylated, while arginine can be mono- and di-methylated [107,108,109]. Histone methylation is commonly observed on the histones H3 and H4. However, H2A and H2B methylations are also noted. Both transcriptional activation and repression are observed with histone methylation. For example, H3K4 and H3K36 methylation was correlated with transcriptional activation, but H3K9, H3K27, and H4K20 methylation had been shown to induce transcriptional repression [110,111]. It was also believed that histone methylation was an irreversible and permanent epigenetic change [112]. However, recently, enzymes such as histone lysine demethylase and arginine deiminase were shown to directly remove a methyl group from lysine residue and antagonize histone arginine methylation, respectively. 

The abnormal loss or gain of histone methylation levels has been demonstrated in tumorigenesis [113]. For example, researchers observed that H2B total methylation levels were increased by treating embryonic cells from *Drosophila melanogaster* with 50 μM trivalent arsenic, whereas H3 and H4 histone methylation were abolished [94,114]. However, mammalian cell responses to arsenic exposure are not simple: the varied effects of As^III^ on the methylation of H3 lysine residues were observed, including higher H3K9 dimethylation (H3K9me2) and H3K4 tri-methylation (H3K4me3) and lower H3K27 tri-methylation (H3K27me3) [115]. A study on human lung carcinoma A549 cells confirmed the increased H3K4me3 after 24-h or seven-day exposures, which is consistent with a previous study that utilized RWPE1 cells initially derived from a human prostate [116,117]. Methylation at H3K4me3 has been shown to correlate with active gene transcription and is generally found in the transcription start sites [118]. However, H3K9me2 is a repression mark. Increased H3K9me2, which is catalyzed by higher levels of G9a protein, a nuclear lysine methyl transferase [115], is associated with reversible modification correlated with transcriptional repression [88], and has been demonstrated to be involved in the silencing of tumor suppressor genes in the cancer cell lines [119,120]. H3K27me3 is also a repressive epigenetic mark that is crucial for regulating genes and the inactivation X chromosome [121]. Although multiple studies have investigated arsenic effects on H3K27me3, the discoveries were inconsistent. A study did not find any alteration of H3K27me3 after treating HepG2 cells derived from a male human liver tumor with 7.5 μM As^III^ [105]. However, Zhou et al. demonstrated that 2.5 and 5 μM μg/L of As^III^ decreased H3K27me3 in A549 cells [115]. This result is consistent with a population-based study among Bangladeshi men in which an inverse association between As exposure and H3K27me3 in peripheral blood mononuclear cells (PBMCs) was observed [122]. In contrast, there was a positive correlation between As exposure and H3K27me3 among women [122], similar to research demonstrating that 0.5 μM As^III^ enhanced this post-transcription modification using embryonic fibroblasts derived from a female mouse [123]. Arsenic also induced epigenetic modification via the generation of oxidative stress [124]. Ma et al. found a positive relationship between arsenic levels in hair and urine, and altered total H3K9me2 and H3K36me3 amounts. The alteration of H3K36me3 was found to be higher in the promoter regions of oxidative stress response (OSR) genes in HaCaT and HEK cells [103]. 

### 3.3. Histone Phosphorylation

The phosphorylation of histone is crucial for chromatin condensation and transcriptional activation during mitosis and meiosis [125]. Phosphorylation can modify all four histone core proteins (H2A, H2B, H3, and H4) and linker protein H1. It mainly occurs on serine, threonine, and tyrosine residues. Phosphatases and kinases control this modification. For example, H2A and H2B phosphorylation is catalyzed by several kinases, such as ataxia telangiectasia mutated (ATM) for H2AX [126]. In contrast, Cyclin-dependent kinases (CDKs) are responsible for H1 phosphorylation [127]. H3 phosphorylation is found during cell cycle progression and regulation of gene expression [128]. Similarly, histone H4 (serine 1) phosphorylation is increased during the cell cycle and is regulated by casein kinase 2 [129]. The function of histone phosphorylation is to loosen the chromatin by acting against the positively charged histone protein [130].

Histone phosphorylation plays an important role in arsenic-induced carcinogenesis. Exposure to a high dose As^III^ (10 μM) has been shown to reduce the total H1 and H3 phosphorylation levels in Chinese hamster ovary cells [131]. However, several studies using various cell lines have demonstrated consistently that different doses and durations of both As^III^ and DMA^III^ can globally induce H3 phosphorylation on a serine residue (H3S10ph) [102,132,133,134,135], which is essential for the regulation of chromosome segregation during mitosis [136]. Studies have also indicated that H3 phosphorylation induced by arsenic exposure may be necessary for the upregulation of the oncogenes c-*fos* and c-*jun* [104] and the induction of caspase 10, a proapoptotic factor [102]. Interestingly, nickel, another metal, has also been demonstrated to increase H3S10 (serine 10) through the activation of the c-jun N-terminal kinase/stress-activated protein kinase (JNK/SAPK) pathway [137]. Arsenic exposure activates JNK and p38/Mpk2 kinase [138], and histone H3 phosphorylation through the JNK/SAPK pathway, which may be a common mechanism of metal-induced histone modification.

Overall, these studies prove that the dysregulation of PTM occurs due to arsenic exposure. However, the findings have been inconsistent in some cases because of many factors, including dose and time of exposure differences, duration and type of arsenic compound, and compounding factors such as sex contribution and measurement error. Hence, further work is necessary to completely unravel the relationship between altered histone modification and arsenic exposure, and to elucidate the total amount of altered PTM of histone on arsenic-induced carcinogenesis and angiogenesis.

## 4. Abnormal Changes of MicroRNAs and lncRNAs upon Arsenic Exposure

### 4.1. MicroRNAs

MicroRNAs (miRNAs) are small non-coding RNAs that participate in different biological regulatory events such as RNA silencing and post-transcriptional regulation of genes. Ambros and colleagues discovered lin-4, the first miRNA in *Caenorhabditis elegans*, as a small non-coding RNA that affected development via regulating the expression of the protein lin-14 [139]. Since then, miRNAs have been found to be present in both invertebrates and vertebrates, and some of them are highly conserved across the species, leading us to believe that miRNA-mediated post-transcriptional regulation is a general regulatory function across species [140,141,142]. Each miRNA is believed to target several hundred mRNAs, and each mRNA may be suppressed by several different types of miRNAs [143]. MicroRNAs regulate mRNA through sequence-specific RNA–RNA interactions in the 3′ untranslated *region* (3′-UTR) of targeted mRNA, destabilizing the mRNA and deactivating gene expression [144,145]. Currently there are 38,589 hairpin precursors and 48,860 mature miRNAs from 271 organisms recorded in miRBase catalogs, though the roles of many miRNAs are still unknown [146]. Approximately 30% of mammalian genes are regulated by miRNAs [147]. In addition, increasing evidence has established the dysregulation of miRNA expression in cell differentiation, proliferation, and angiogenesis that can lead to carcinogenesis via different mechanisms. These mechanisms include amplification or deletion, a transcriptional control of miRNAs, dysregulated epigenetic changes, and defects in the miRNA biogenesis machinery [145].

It has been demonstrated that miRNAs are heavily dysregulated in cancers [19,148]. Because miRNAs are negative regulators of gene expression, dysregulation of these miRNAs can be tumorigenic if targeted mRNAs are either tumor suppressors or oncogenes. For instance, the let-7 miRNA family directly targets the *RAS* oncogene to suppress its expression, and the reduction of let-7 miRNA family members leads to the overexpression of RAS oncoprotein. Conversely, perturbation of the miR-34 family leads to the dysregulation of the p53 tumor suppressor pathway [40,149].

Although notable progress has been made regarding the biogenesis and mechanisms of miRNAs in different types of cancer, our knowledge is limited about the dysregulation of miRNAs in As-induced carcinogenesis. The very first study of arsenic-induced miRNA dysregulation in cell transformation was done by Wang et al. in 2011. They found that exposure to a low concentration of arsenic for 16 weeks led to malignant transformation and reduced miR-200b/c expression in immortalized human bronchial epithelial cells (HBECs) with p53 knockdown. The inhibition of malignant transformation occurred when the same cells were forcefully expressed with miR-200b [150]. Since then, there has been growing evidence of miRNA dysregulation in As-mediated carcinogenesis (Table 2).

Kong et al. found an association between the reduced level of miR-21 and an increase in urinary arsenic levels in Hong Kong children aged 12–19 [170]. In contrast, there was an increase of miR-21 and miR-222 in the peripheral blood of steelworkers [171]. When non-malignant human keratinocytes (HaCaT) were treated with arsenic, 30 miRNAs were differentially expressed in arsenic-exposed cells compared to control cells. This study confirmed the upregulation of previously found miRNAs involved in carcinogeneses, such as miR-21, miR-200a, and miR-141, which were indicated as potential biomarkers for the epithelial phenotype of cancer cells [172]. In addition, exposure to As led to the upregulation of miR-151 and miR-183 in liver tissues of rats [173] and miR-155 in cultured 16-HBE cells [158]. Zeng et al. evaluated the relationship between the expression of miRNAs and multiorgan damage in control and arsenic-exposed populations in China [155]. The study found associations between miR-155 and arsenic-induced skin damage between miR-21, miR-145, and liver damage, and between miR-191 and kidney damage, indicating that these miRNAs act as potential biomarkers for As-induced multiorgan injury. In a recent study, Al-Eryani et al. analyzed the miRNA expression profile in non-malignant hyperkeratosis (HK) and malignant skin lesion tissues, squamous cell carcinoma (SCC), and basal cell carcinoma (BCC) from West Bengal (India) people chronically exposed to high levels of arsenic, and found the differential expression of 35 miRNAs among the three skin lesions. They found that miR-425-5p and miR-433 were upregulated in both BCC and SCC compared to HK and were potentially associated with malignancy. However, miR-184 and miR-576-3p were upregulated in SCC alone compared to both BCC and HK. MiR-29c, miR-381, miR-452, miR-487b, miR-494, and miR-590-5p were selectively decreased in BCC compared to both SCC and HK. They summarized both phenotype- and stage-related differential miRNA expression profiles that may serve as possible biomarkers for arsenic-induced internal tumors [9,152].

Several potential mechanisms are associated with miRNA dysregulation in As-mediated cancerous outcomes. For example, As exposure led to the generation of reactive oxygen species (ROS) and conceivably changed the miRNA expression [41,163,164]. A study found that miR-21 was upregulated in the malignant transformed human embryo lung fibroblast (HELF) cells after As exposure, which was due to the activation of the ERK/NF-κB pathway by ROS [174]. We also showed that chronic As exposure led to ROS generation in human bronchial epithelial BEAS-2B cells, which induced cyclooxygenase-2 (COX2) and hypoxia-inducible factor (HIF)1-α expression through miR199a-5p suppression, thus promoting tumor growth and angiogenesis. The forced expression of miR-199a-5p suppressed COX2 and HIF1-α expression and impaired arsenic-induced angiogenesis and tumor growth [153]. Another mechanism study showed that arsenic promoted epithelial-mesenchymal transition (EMT) by inducing pro-inflammatory cytokine interleukin-6 (IL-6) secretion, mediating the signal transducer and activator of transcription 3 (STAT3) signaling, and increasing miR-21 expression in an autocrine manner [175]. In addition, miR-301a was also found to be increased in human lung epithelial BEAS-2B cells exposed to As, and miR-301 was an oncogenic miRNA that directly antagonized SMAD4 in the IL6/STAT3/miR-301a/SMAD4 signaling pathway during As-induced carcinogenesis [151]. Liu et al. found that increased miR-21 expression inhibited tumor suppressor programmed cell death protein 4 (PDCD4) and activated the ERK signaling pathway through the decreased expression of tumor suppressor PTEN [176]. Similarly, the induction of miR-222 expression by As exposure inhibited PTEN expression and was responsible for inducing cell transformation and tumor growth [164].

### 4.2. Long Noncoding RNAs (ln cRNAs)

Long noncoding RNA (lncRNA) is a type of RNA that is greater than 200 nucleotides and is not translated into a protein. Like mRNAs, lncRNAs are also generally transcribed by RNA polymerase II and processed with a 5′-cap structure and 3′-end poly-A, followed by RNA splicing and editing to create isoform transcripts. Because of their low expressions, lncRNAs were primarily thought to be a transcription noise. However, with the advancement of technology and better understanding, lncRNAs were observed to be involved in the transcription and post-transcription regulation via interaction with RNA, DNA, or proteins [177]. The lncRNAs can be found in the genomic loci, which are putatively intronic, intergenic, or intersected with protein-coding regions in either sense or antisense orientation, which can control the target gene expression in the downstream via cis- or trans-regulatory mechanism. In addition, lncRNAs also regulate mRNA splicing and act as predecessors to noncoding RNAs (ncRNAs), such as miRNAs [178,179,180]. They can function as tumor suppressors or oncogenes and play roles in various signaling pathways [181]. Several lncRNAs have been recognized as independent or additional biomarkers in the diagnosis and prognosis of cancer [182].

A recent study found that programmed cell death 1 ligand (PD-L1) and STAT3 were upregulated in arsenic-transformed BEAS-2B cells, and knockdown of STAT3 inhibited arsenic-induced PD-L1 upregulation. Lnc-DC, an lncRNA, was an upstream regulator to mediate arsenic-induced STAT3 activation, suggesting that Lnc-DC/STAT3 cascade may mediate PD-L1 upregulation during arsenic-induced transformation [154]. In lung cancer cells, STAT3 is directly bound to the PD-L1 promotor and is necessary for PD-L1 expression [183,184]. Ji et al. found that the expression levels of metastasis-associated lung adenocarcinoma transcript 1 (MALAT1), one of the well-known lncRNAs, highly correlated with the tumor stage and metastasis of non-small cell lung cancer (NSCLC) [185]. In the area of arsenic exposure, the levels of MALAT1 expression were increased in hepatocellular carcinoma (HCC) patients [186]. Increased hypoxia-inducible factor 2α (HIF-2α) and MALAT1 expression levels were also found in HCC tissues and arsenic-induced transformed human hepatic epithelial (L-02) cells. Functionally, the upregulation of MALAT1 and HIF-2α enhanced the invasive capability of arsenic-transformed L-02 cells and HCC-LM3 cells. Mechanistically, As induced MALAT1 and separated the von Hippel-Lindau (VHL) protein from HIF-2α to reduce the ubiquitination of VHL-mediated HIF-2α, resulting in HIF-2α accumulation. In L-02 cells, arsenite exposure enhanced glycolysis [187]. In addition to HIF-2α upregulation as above, arsenic exposure also increased the expression of HIF-1α through the lncRNA MALAT1. Furthermore, arsenic exposure enhanced glycolysis by HIF-1α stabilization via MALAT1, but not by HIF-2α [187]. Currently, it is well established that enhanced glycolysis plays an essential role in cancer initiation and progression [188,189,190]. These discoveries give additional proof that supports a critical role of the MALAT1 upregulation in arsenic-mediated carcinogenesis. Animals exposed to arsenic treatment also showed the upregulation of MALAT1 during the progression of mouse liver fibrosis. Together, these studies show that arsenic exposure upregulates MALAT1 expression in both cultured cells and mice, suggesting a critical role of lncRNA MALAT1 in arsenic carcinogenicity and toxicity [191]. However, the role and mechanisms of these upregulated lncRNAs in arsenic-induced carcinogenesis are currently not clear and remain to be elucidated.

Overall, arsenic exposure changes the expression profiles of miRNAs and lncRNAs, which may serve as potential biological markers and provide therapeutic values for arsenic-induced carcinogenesis. However, the research is limited to lncRNAs, and more studies are necessary to unravel their function during this process. Similarly, studies are also required to investigate miRNAs in As-induced cancer in different stages, especially mechanisms related to direct vs. indirect effects of arsenic-targeted miRNAs on the population and how these miRNAs control different signaling pathways to cause cancer and other diseases.

## 5. Arsenic Causes Abnormal RNA Modification

RNA methylation is a reversible post-transcriptional alteration to RNA that epigenetically regulates different biological processes and is widely present in both eukaryotes and prokaryotes [192]. In this process, a methyl group is transferred from an active methyl compound to a different compound. It occurs not only in messenger RNA (mRNA), but also in other RNA species, including transfer RNA (tRNA), ribosomal RNA (rRNA), transfer-messenger RNA (tmRNA), small nucleolar (snoRNA), microRNA, viral RNA, and so on [193,194]. RNA methylation modulates RNA splicing [195], stability [196], translation [197,198], DNA damage repair [199], nuclear export [200], miRNA biogenesis initiation [201], immunogenicity [202], and the occurrence and development of cancer [203,204]. Among more than 170 types of modification that have been observed in all kinds of RNAs, methylation accounts for more than 50% of them. When methylation is found at the sixth N of the adenylate of RNA, it is called m6A methylation. Studies also found other forms of RNA methylation besides m6A methylation, such as m6A_m_, m7G, m1A, and m5c. However, m6A alteration has been considered the most abundant methylation alteration in the eukaryote mRNA [205] that affects every process in the life cycle of RNA [206].

M6A methyl transferases such as methyltransferase-like enzyme 3/14 (METTL3/14), Wilms tumor 1-associated protein (WTAP), RBM15/15B, and KIAA1429 catalyze m6A modification [207]. The binding proteins, called ‘readers’, which can recognize and bind to the methylated RNA, decode m6A methylation, and generate a functional signal. Readers include eukaryotic initiation factor (eIF) 3, YT521-B homology (YTH) domain-containing protein [208], heterogeneous nuclear ribonucleoprotein (HNRNP) protein family [209], and the IGF2 mRNA binding proteins (IGF2BP) family [210]. On the other hand, demethylases such as fat mass and obesity-associated protein (FTO) and alkB homolog 5 (ALKBH5) are called “erasers” because they remove the methyl group from the target mRNAs. Growing evidence has suggested that the affluence of m6A and expressions of its regulators, including writers, erasers, and readers, are often dysregulated in different types of cancers and are essential for cancer initiation, progression, metastasis, as well as drug resistance and cancer relapse [211,212,213,214,215]. For instance, METTL3 can recruit translation initiation factors directly and increase RNA translation. It promoted cell growth, survival, and invasion by upregulating EGFR and TAZ in lung adenocarcinoma [216]. Choe et al. demonstrated that METTL3 promoted the translation and transformation of oncogenes and formed dense polyribosomes by interacting with EIF3H in primary lung cancer, which might be a potential therapeutic target [217]. The role of METTL14 in lung cancer is controversial; a recent study showed that METTL14 knockdown suppressed the malignant progression of non-small cell lung cancer (NSCLC) by reducing Twist expression [218]. However, other studies showed that METTL14 was downregulated in lung adenocarcinoma (LUAD) and mediated lncRNA HCG11 [219] or miR-30c-1-3p to inhibit tumor growth [220]. The overexpression of FTO decreased m6A levels in MZF1 mRNA transcripts, increased mRNA stability, and promoted MZF1 expression, leading to the proliferation and invasion of lung squamous cell carcinoma cells [221]. Although m6A RNA modification dysregulation is associated with various cancers, the underlying mechanisms of m6A in cancer have not yet been fully understood.

Gao et al. found that As treatment reduced m6A modification near the stop codon of an endogenous inducer of somatic mutation gene APOBEC3B (A3B) in the human alveolar basal epithelial cells from adenocarcinoma. FTO was responsible for reducing m6A alteration in A3B, which led to increased A3B expression and higher DNA mutation rates of the m6A reader YTHDF2. They confirmed that A3B was a downstream target of FTO in lung tissues from As-exposed mice. FTO protein expression was positively correlated with A3B protein expression in tumor samples from human NSCLC patients [222]. Another study also confirmed that As treatment increased FTO expression, decreased m6A RNA methylation, and consequently induced malignant transformation and tumorigenesis in keratinocytes. FTO deletion inhibited arsenic-induced tumorigenesis in both in vitro and in vivo experiments. Arsenic stabilization of the FTO protein occurred via impeding p62-mediated autophagy, which led to a positive feedback loop to keep up FTO accumulation [223]. Unfortunately, few studies have been performed on As-induced dysregulation of m6A methylation; hence, further studies are necessary to understand the potential molecular mechanisms of m6A in As-induced tumorigenesis and cancer progression.

## 6. Arsenic Exposure and Alternative Splicing

The removal of intronic sequences and splicing together of adjacent exons are necessary for RNA maturation and translation into protein. The elimination of introns is followed by attaching exons in their DNA-corresponding order, known as consecutive splicing, which occurs at every intron-exon boundary [224]. Alternative splicing diverges from this process via mechanisms that reorder the pattern of exons into alternative coding sequences that translate to different proteins. This mechanism is an evolutionarily conserved process that significantly increases transcriptome and proteome diversity from a limited genome. Alternative splicing is necessary to maintain cellular homeostasis and is essential in regulating cell differentiation and development [225,226]. Alternative splicing is tightly controlled by other significant processes in the cell, and perturbation of this process is known to occur commonly in human cancers [63,227,228]. Alternative splicing is associated with carcinogenesis [229], angiogenesis [230], and EMT [227,228]. Growing evidence has demonstrated that the decision of alternative splicing or consecutive splicing occurs while the mRNA is still tied to the DNA and takes place transcriptionally [231]. Several studies have shown the factors that control the structure of chromatin, such as histone PTMs and DNA methylation, dictate the selection of exon candidates for splicing [232,233,234].

As discussed, exposure to iAs significantly changes DNA methylation and histone post-translational modifications. Hence, it is reasonable to believe that it may play a part in alternative splicing by changing chromatin organization. Cardoso et al. found that when human immortalized human keratinocytes (HaCaT) were treated with sodium arsenite at 100 nM for 28 weeks, a minimum of 600 different alternative splicing events were found at each time point tested. They found that chronic arsenic exposure induced the canonical isoforms of the splice regulators DDX42, RMB25, and SRRM2 [235]. Alternating splicing might occur via DNA binding inhibition by alternative splicing modifiers such as CCCTC-binding factor (CTCF), TET1/2, and poly (ADP) ribose polymerase (PARP1) [64]. Several other studies also showed that PARP-1 inhibition occurred in arsenite-exposed cells [236,237], and PARP-1 was a direct molecular target of arsenite, which selectively interacted with zinc finger domains [236,237,238]. Notably, many splicing factors are regulated by PARylation [239,240,241], and inhibition of PARP1 binding to DNA upon arsenic exposure affects the structural properties of chromatin and the PARylation activities, which indirectly controls splicing decisions. iAs also inhibits the methylcytosine dioxygenases (TET1/2), the DNA-binding proteins with zinc finger motifs. TET1/2 are necessary to oxidize 5-methylcytosine to 5-hydroxymethylcytosine and 5-carboxylcytosine [242]. Deactivation of TET1/2 allows 5-methylcytosine to assemble at the CTCF target sites and stops CTCF from attaching to its target sites, consequently leading to exon exclusion [243]. Thus, iAs may participate in splicing decisions by blocking the binding of PARP1 or CTCF to DNA.

iAs also changes alternate splicing by upregulating p52 via non-canonical NF-kB pathway activation [244]. p52 modulates the splicing factor SRSF1 by co-localizing and interacting with it [245]. SRSF1 overexpression is induced by MYC [246], which is also dysregulated in iAs exposure [53,247]. MYC increases core pre-mRNA machinery in the process of carcinogenesis and maintains the suitable splicing of alternative exons [248]. More studies are needed to further elucidate how iAs dictates alternating splicing.

## 7. Conclusions and Future Direction

Arsenic alone is not efficient to cause point mutation or initiate and promote tumor development in animal models. However, growing evidence has shown that arsenic causes the dysregulation of epigenetic changes, including DNA methylation, histone modification, miRNAs and lncRNAs, RNA modification, and alternative splicing, which consequently changes the gene expressions followed by severe pathologies, including cancers. Our understanding of the underlying epigenetic mechanisms is still limited, especially for RNA methylation, lncRNAs, and alternative splicing. More studies are necessary to elucidate their roles and mechanisms in arsenic-induced carcinogenesis. In addition, most studies used different cell lines and animals to characterize epigenetic changes induced by arsenic exposure. More population studies using human cohorts exposed to varying arsenic levels are necessary to unveil how individual variability, genetic background, and other confounding variables such as diet, gender, and age may influence the epigenetic responses. Studies are also required to systematically investigate epigenetic profiles to identify and validate the markers of epigenetic changes in targeted disease-relevant tissues such as the skin, bladder, kidney, and lung.

In summary, a comprehensive epigenomic approach is necessary to understand the mechanisms of arsenic-induced carcinogenesis and angiogenesis. These mechanistic comprehensions of epigenetic changes can provide potential biomarkers of arsenic exposure and develop potential therapeutic targets for mitigating the global burden of arsenic-induced diseases, including cancers.

## Figures and Tables

**Table 2:** miRNA alteration and carcinogenesis due to arsenic exposure.

MicroRNAs	Biological Samples	Alteration	Target Genes & Function	References
miR-21	HELF, Human Bronchial Epithelial (HBE), and human umbilical vein endothelial cells (HUVEC)	Up	ROS activation of ERK/NF-kb pathway EMT transition by acting on PTEN and PCD4 Enhanced levels of vascular endothelial growth factor (VEGF) to increase angiogenesis	[151,152,153,154]
	A urine sample from Hong Kong children	Down	Not known	
	Blood plasma from the Chinese and Indian population	Up	Association with liver damage	[155,156]
miR-145	Blood plasma from the Chinese population	Up	Indicated impact on immune inflammation, oxidative stress, and DNA repair mechanisms Association liver damage	[155,157]
miR-155	Blood plasma from the Chinese population	Up	Association with skin damage	[155]
	HBE cells	Up	miR-155 induced cell malignant transformation by targeting Nrf2-mediated oxidative damage	[158]
miR-190	Human lung epithelial cells	Up	Activate Akt signaling via downregulating PHLPP Promote angiogenesis through increasing VEGF expression	[159]
miR-191	human bronchial epithelial (HBE) cells	Up	HIF-2α increased Wilms’ tumor 1 (WT1) *via* miR-191 involved in the angiogenesis and metastasis of Transformed-HBE cells	[160]
	Blood plasma from the Chinese population	Up	Association with kidney damage	[155]
miR-222	Hepatocellular carcinoma	Up	Inhibition of apoptosis by regulating different target such as p27, TIMFE and FTEN	[161,162,163]
	Arsenic-induced BEAS-2B (As-T-cells)	Up	Inhibition of apoptosis by regulating target FTEN	[164]
miR-301a	Arsenic-induced BEAS-2B (As-Tcells) and Xenografts model	Up	Malignant transformation of BEAS-2B cells by acting on directly SMAD4 via STAT3/miR-301a/SMAD4 Loop	[151]
miR-425-5p and miR-433	Premalignant and malignant skin tissue from an Indian population	Up	Association with hyperkeratosis that leads to conclude their association with malignancy	[152]
miR23a, miR-27a, miR-122, miR-124, and miR-126	Blood plasma from the Indian population	Up	Association with skin lesions	[165]
miR-1282 and miR-4530		Down		
miR-199a-5p	Arsenic-induced BEAS-2B (As-T-cells)	Down	Upregulate HIF-1 alpha and COX-2 to promote angiogenesis	[153]
miR-200 b	Immortalized p53-knocked down HBE	Down	Increased expression of ZEB1 and ZEB2, which are EMT-inducing transcription factors	[150]
miR-9	In vivo experiment on the fertilized egg	Down	Increased NRP1 transmembrane receptor to promote vascular development	[166,167]
miR-181b	In vivo experiment on the fertilized egg	Down	Increased NRP1 transmembrane receptor via miR-181b downregulation to promote vascular development	[166,167]
miR-182-5p	Human retinal epithelial cells	Down	Increased HIF2α through miR-182-5p suppression contributed to arsenic-induced malignant transformation of human renal epithelial cells.	[168]
miR-31	BEAS-2B cells	Down	arsenic induces malignant transformation of BEAS-2B cells by the overexpressing SATB2 and inhibiting miR-31 expression	[169]
miR-126	Blood plasma from the Indian population	Down	Precancerous and cancerous skin lesions	[165]

## Data Availability

The data presented in this study are openly available in Medline and Embase.
